# Differential Surgery on the Rheumatoid Wrist: Patient Satisfaction and Clinical Outcome

**DOI:** 10.3390/medicina62071400

**Published:** 2026-07-20

**Authors:** Christoph Biehl, Madita Biehl, Lotta Biehl

**Affiliations:** 1 Department of Trauma, Hand and Reconstructive Surgery, University Hospital Giessen-Marburg GmbH, Giessen Campus, Rudolf-Buchheim-Strasse 7, 35392 Giessen, Germany; 2 University Medical Center, Johannes Gutenberg University Main, 55131 Mainz, Germany; madita.biehl@gmx.de; 3 Heidelberg Faculty of Medicine, Ruprecht-Karls-University Heidelberg, 69115 Heidelberg, Germany; lotta.biehl@gmx.de

**Keywords:** rheumatoid arthritis, rheumatoid wrist, radio-lunate arthrodesis, wrist prosthesis, PROMs, stage-adapted operation, differential surgery

## Abstract

*Background and Objectives*: The wrist is one of the most common sites of rheumatic disease. Based on the graded deformities of Larsen et al., surgery has been adapted to minimize harm while maximizing benefit to the individual. This study aims to survey the institutional experience, describing functional outcomes and patient satisfaction across several surgical approaches, without formal statistical comparison. *Materials and Methods*: The studies and results presented here are from one center specialized in rheumaorthopedic surgery and reflect the above concepts. The studies cover a broad spectrum of surgical techniques, which were categorized according to the Larsen stages of wrist destruction. All studies were retrospective or retrospective-like, and follow-up data were analyzed collectively. The primary endpoints were functional outcome scales and patient satisfaction, supplemented by pain relief, as well as strength and range of motion. The assessment utilized, amongst others, the QuickDASH, FFbH and Clayton score, as well as appropriate patient-reported measures such as ADL and SF-36. Radiological follow-ups were classified according to the Larsen–Dale–Eek classification; for endoprostheses/arthrodesis, carpal height was determined according to Youme. *Results*: Significant improvements in the Clayton score (consistently greater postoperatively than preoperatively) are evident for several procedures, particularly MPW^®^ (+37 points) and APW^®^ (+28 points). Postoperative reductions in VAS pain scores are marked for most procedures, e.g., MPW^®^ from 7 to 1.8. The revision rates for most procedures are around 10–13% during the follow-up periods. In addition, the tendon and soft tissue functions of the hand(s) play critical roles in these PROMs. *Conclusions*: The studies present the results and limitations of surgical treatment for rheumatic wrists in the prebiological era. The results of individual studies need to be examined more closely to better assess the potential and limitations of surgery for patients.

## 1. Introduction

In rheumatic disease, the wrists are almost affected during the disease course. The introduction of biologics and earlier diagnosis confirmation has improved the long-term prognosis, which is also reflected in the increased demands of patients, especially for hand function. Early arthrodesis of the wrist, which was common in the past, significantly limits the ability to use the hand.

**Figure 1:**
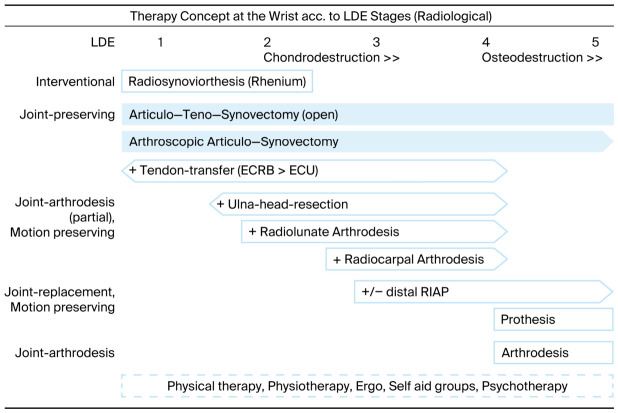
Therapy concept at the wrist according to LDE stage. Overview of the different surgical treatment options for inflamed joints. ECRB = extensor carpi radialis brevis, ECU = extensor carpi ulnaris, RIAP = resection-interposition arthroplasty.

If surgical treatment is needed, preservation of residual mobility must be included in planning, with the condition of the bone, reconstructability of the carpal height, soft tissue balance, and kinematics of the hand carefully examined in advance [1,2,3]. Recent developments in wrist arthroplasty have renewed interest in motion-preserving treatment even in advanced rheumatoid destruction, although complication rates and bone quality remain important concerns [4]. For rheumatoid patients, the precarious bone situation with early-onset osteoporosis and the associated bone mass loss is compounded by concomitant pathologies of the periarticular tissues, especially tendons and ligaments [5,6]. With the widespread adoption of biologic agents as first-line therapy, treatments for the rheumatoid hand, which were reserved exclusively for arthritically altered joints, are gaining ground. A question is whether patient satisfaction after surgery for the rheumatoid wrist is determined primarily by pain relief and radiographic stabilization, or whether it depends more strongly on restoration of global hand function, particularly extensor tendon function. This issue is clinically relevant because extensor tendon damage may progress slowly, may be compensated for over long periods, and may be underestimated until partial or complete rupture has occurred [7,8]. Once tendon function is lost, improvement in pain alone may not be sufficient to restore useful hand function. Therefore, the primary question of this study was whether postoperative patient satisfaction and functional outcomes after surgical treatment correlate with overall hand function. We hypothesized that patients with preserved or successfully reconstructed wrist and tendon function may report better functional outcomes and higher satisfaction than patients with persistent or progressive tendon dysfunction. The aim of this study was to summarize retrospective studies of stage-adapted rheumatoid wrist surgery, including joint-preserving procedures, partial arthrodesis, and wrist arthroplasty. Because of the heterogeneity of the available studies, this hypothesis was assessed descriptively rather than by formal pooled statistical analysis (Figure 1).

## 2. Materials and Methods

More than 1600 operations on rheumatoid wrists were performed at the Center for Orthopedic Rheumatology between 1984 and 2015. The patients included in the studies had developed RA before the year 2000, and even after that, only a small percentage of them received bDMARD therapy. The indications for surgical treatment of the wrist are refractory arthritis and periarticular inflammation with concomitant pathologies and limitations of function. The various surgical procedures were primarily guided by the staging of radiological changes and intraoperative findings in the hand and extensor tendons (Figure 1).

For this review study, various previously published institutional studies only from the center specializing in rheumaorthopedic surgery, covering a range of surgical techniques according to the Larsen stage of wrist destruction, were re-evaluated and compared with each other. The inclusion criteria for the included studies were defined primarily in terms of long-term follow-up periods of at least 10 years, and patient recruitment was organized accordingly. These studies were summarized regarding functional outcomes and patient satisfaction with therapy. All patients who take place in follow-up provided written informed consent to participate. Ethical approval was not required, with the exception of the study on MPW^®^ prostheses (Waldemar Link GmbH & Co. KG, Hamburg, Germany), as these studies—being designed as retrospective studies—only had to be reported to the ethics committee (Regional Ethics Committee). The study on MPW^®^ prosthesis was approved by the ethics board (Regional Ethical Review Board No. 837.157.14 (9396-F)). The ethics approvals relate to the individual studies; no ethics approval was required for this descriptive synthesis. During the follow-up examinations, the parameters of pain, strength, range of motion, and daily functions were assessed.

The range of scores represents the different aspects of the impairment of function of the affected wrist to the whole extremity (Quick DASH) and the overall situation of the patient (SF-36; FFbH) [1,9]. The Clayton score also allows evaluation of extensor tendons by considering the inflammatory alterations at the wrist. This is the only score that provides a physician’s measurement tool for wrist function; the other scores are based on information provided by the patient.

The different grip positions and assessments of grip strength were recorded using the ADL and FFbH scores [3], which go beyond the pure recording of hand function and focus on self-care and mobility. The widespread DASH score also focuses on the recording of functional limitations, additionally differentiating sensitive limitations (pain, dysesthesias, psychosocial issues) from motor entities, and is limited primarily to the affected extremity. The SF-36 allows comparisons with health surveys [4]. The radiological controls considered rheumatological staging according to Larsen, Dale, and Eek [10].

### Statistical Analysis

This study is purely descriptive; therefore, no formal meta-analysis of the data was carried out. The comparative analyses were descriptive. Therefore, the focus was placed on discussing the variability in the results of the included studies. Only the descriptive and scoring results from the publications were compared. There was no a priori or post hoc power analysis, because the number of patients included in the studies was determined primarily by the surgical procedure and the average duration of follow-up. Apart from our own work, no statistical analysis had been carried out. In our study, for radio-carpal arthrodesis, we use a two-way ANOVA test and a Mann–Whitney U test for non-parametric samples (see Section 3.4). If available, *p*-values of post hoc tests were adjusted for multiple comparisons. The level of significance therefore was set at <0.05 for all analyses.

## 3. Results

The rheumatoid wrists treated surgically at the center specializing in rheumaorthopedic surgery were predominantly joint-preserving (approx. 75%; total arthrodesis, 7%; prostheses, 15%). The studies do not all use identical scores. As early as 1984, patients were asked about their limitations in daily life via a simplified score for daily functions (including FFbH) and a modified 100-point Clayton score (mobility, stability, ligament tension, and pain (4-point Likert scale)) pre- and postoperatively. The latter studies additionally used Quick DASH (Table 1). The radiological follow-up controls were classified according to the stages according to Larsen et al. or, in the case of endoprosthetic treatment or arthrodesis, the carpal height was determined according to Youm [11].

### 3.1. Articulo-Teno-Synovectomy

In 2000, long-term results of wrist synovectomy were presented at national congresses (Southern German Congress for Orthopedic and Traumatology, German Congress for Orthopedic and Traumatology) [5]. A total of 110 operations in 89 patients with a confirmed RA diagnosis were performed. At the time of follow-up, 78 patients with 88 operations were included, 7 patients died, and 4 patients’ vital signs were unknown. The mean age at surgery was 51.6 years, the duration of the disease was 6.6 years, and the wrist was affected for a mean of 5.1 years. Pain improved from 7.6 to 3 on the VAS, with improvements primarily in daily living capacity and rest pain. The daily function score improved by an average of 4 points from 18.8 to 22.8, and only seven patients were not satisfied with the long-term outcome. For 13% of the wrists, further movement-restricting surgery was required during radiological progression.

### 3.2. Articulo-Teno-Synovectomy with Tendon Transfer

In 1996, Dinges and Thabe reported the results of combined synovectomy with tendon transfer from the 1/2 ECRB to the ECU [6]. From 1988 to 1992, 64 patients underwent this procedure, 59 operated wrists additionally underwent resection of the ulnar head, and 56 patients were followed up for an average of 46.8 months (15 to 72 months). The average age at surgery was 55 years, and the average duration of disease was 7.9 years, with all patients suffering from chronic rheumatoid arthritis. The parameters of swelling, pain, strength, and subjective patient judgment were evaluated, with patients exhibiting little swelling and pain usually showing an improvement in strength, and with a high level of satisfaction being shown. Moreover, a recurrence of swelling was observed in two patients, which correlated with significant pain and a loss of strength, which the patients acknowledged with a subjectively poor result.

### 3.3. Radio-Lunate Arthrodesis

Schill et al. were able to follow up 50 patients with 57 wrists for an average of 7 years after surgery in their retrospective study in 2002 [12]; a total of 69 surgeries were performed between 1988 and 1994. The mean age at surgery was 54.4 years, and the mean duration of disease was 9.6 years. At follow-up, the Clayton score and questions about daily function were surveyed. Pain was recorded as part of the Clayton score within a 4-point Likert scale, as it had been during synovectomy with tendon transfer. The Clayton score improved to 74.2 points, although the preoperative score was not reported. Satisfied patients scored 18.3 points in the daily life score, whereas those who were dissatisfied with surgery scored only 8.3 points. The VAS score decreased from 7.6 points preoperatively to 4.2 points postoperatively, and the revision rate was approximately 8%.

### 3.4. Radiocarpal Arthrodesis with Resection-Interposition Arthroplasty

In the 2018 study, 28 patients with 34 wrists were followed up [13]. From 1985 to 2007, a total of 59 operations were performed in 50 patients with RA. Five patients died at time of follow-up and 17 were unknowingly deceased. The mean age was 60.6 years, the mean duration of disease was 18.2 years, and the median follow-up time was 9 years. Outcomes were assessed via various scores (Clayton, Quick DASH, Functional Questionnaire, VAS). The Clayton score improved from 45 points preoperatively to 68 points postoperatively, while the daily life score increased from 22 points preoperatively to an average of 26 points postoperatively.

### 3.5. Prosthetics (Swanson, APW^®^, MPW^®^)

Over the years, various wrist prostheses have been implanted at the Center specialized for Orthopedic Rheumatology. From 1984 to 1992, 102 silastic wrist prostheses were implanted in patients with RA in LDE stages 4 and 5. The prosthesis models were followed up, and the results were published. Long-term results of the Swanson prosthesis (Styker, Portage, MI 49002, USA) of the wrist were obtained by Schill et al. in 2001, and they were able to include 72 patients with 82 prostheses [14]. The peak age was 57 years, the mean duration of the disease was approximately 16 years, and the mean duration of symptomatic wrist disease was 11.8 years. Data collected at follow-up were evaluated with the Clayton score, VAS score, and daily function score.

From 1993 to 1999, 46 APW^®^ prostheses (Waldemar Link GmbH & Co. KG, Hamburg, Germany) were implanted in 39 rheumatic patients. In 2003, Schill and Thabe published their follow-up study [15]. They were able to document 37 patients in a prospective study with a mean follow-up time of 4.6 years. Two patients died. Again, the Clayton score and the VAS served as a basis. Within this short period, 7 of these 39 prostheses had to be revised due to material problems, which led to further development of the prosthesis [15].

In our 2021 study “Long-Term Results of the Modular Physiological Wrist Prosthesis (MPW^®^)”, the successor model also did not yield good results in terms of the scores used (Clayton, Quick DASH, VAS) or patient satisfaction [16]. The second generation of this type of prosthesis was implanted a total of 62 times between 1998 and 2015. We included 32 patients with 34 endoprosthesis-supplied wrists in the study, as we wanted to ensure a follow-up period of at least 8 years. Eight patients had died at time of follow-up, and eight were unknown, possibly deceased. The mean age at the time of surgery was 56 years, and the mean duration of the disease was 18 years. The period between diagnosis and primary implantation was 16.4 years and follow-up was an average of 8.5 years postoperatively. The VAS improved from 7 points preoperatively to 1.8 points postoperatively, and the average DASH score was 47.1 points postoperatively, which corresponds to a poor result, but there is a pronounced spread from 1.7 to 88.8 points. The Clayton score increased from 49.3 preoperatively up to 86.4 postoperatively.

### 3.6. Tendon Reconstructions

In a publication on the reconstruction of tendon lesions at the level of the wrist (levels 6 and 7 acc. to Verdan) [17], 26 patients were followed up, with a total of 27 operations out of an original 62 procedures in 41 patients. Data collected at follow-up were evaluated with different scores (Clayton, Quick DASH, FFbH (only postop value), and VAS). Pain improved from 7.5 points preoperatively to 2.9 points postoperatively, and the subjective patient ratings (PROMs) of general daily function differed little from the preoperative values. Likewise, it is used for assessing subjective strength. However, tendon function was rated better than overall hand function. Revision interventions were not required [18].

## 4. Discussion

Recommendations for the surgical management of inflammatory wrists are based on data and experience from local centers, which, in turn, are mostly based on older publications from the prebiological era. Publications comparing different surgeries in the context of a syllabus on corresponding changes in the rheumatoid wrist are rare.

**Figure 2:**
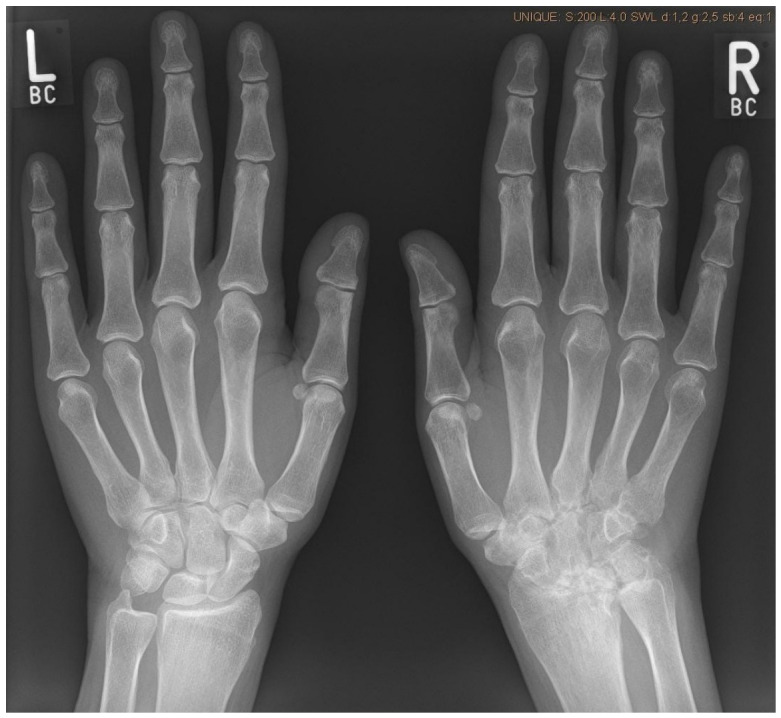
X-ray of both hands. Different stages of destruction of the carpal row between the right and left sides. © C. Biehl.

According to the scoring system of Larsen, Dale, and Eek, radiological follow-up is based on staging (Figure 2) [8]. It is not helpful for endoprosthetic joints or arthrodesis, because the joint surfaces are not assessed. However, determining carpal height provides more information, as it allows comparisons with joint-preserving interventions [18]. With the approval of biologics, limitations arise for the clinical statement because clinical progression is no longer directly coupled with radiological changes.

The inconsistent use of different scores and unclean terminology, particularly in Schill et al., complicates the comparability of study results [9]. In early studies, evaluation was mostly based on subjective assessments, pain indications, and secondary radiological changes, as objective scores were missing. Until the early 2000s, differential therapy and patient recommendations were derived from these evaluations. The introduction of biologics and their increasing use in basic RA therapy have fundamentally changed the situation. Patients’ demands on their joints and overall quality of life increase as systemic disease activity decreases. Currently, mostly isolated tendons are addressed in the hand, and arthroscopic surgery, including assisted (partial) arthrodesis, is increasingly used on the wrist itself, as it is in patients with osteoarthritis. In addition, individual patients continue to present with severe disease as they did 30 years ago. Because of the limited comparability, future studies should collect data using multiple scores. A single score does not adequately reflect the real situation, as not all items can be included in one score. We recommend the use of a combination of Quick DASH, Clayton, ADL and VAS. The individual scores have a certain average weighting, but each has a different emphasis. When assessing radiological changes, the Larsen classification has its limitations when the actual joint line has been altered by prostheses or arthrodesis. Again, radiographic changes should be assessed independently of the joint situation, e.g., using the Youm height index. To improve comparability, it would be useful and desirable to follow all patients in the above trials. However, over the long course of the trials, many patients died or required further hand surgery, so long-term observation with small numbers of patients and limited statistical power must be viewed critically.

However, despite improved early diagnosis and advances in drug therapy, surgical intervention targeting the destroyed articular and periarticular structures may still benefit “rebellious” joints that show persistent inflammation. At an early stage, the inflammatory process can spread to the tendons due to the spatial situation of the periarticular structures, resulting in detectable changes [19,20]. Extensor tendon ruptures occur more frequently at mechanically stressed sites, such as the Lister’s tuberosity or over the ulnar styloid. Patients can tolerate long-term articular and tendon pathologies if they occur slowly and with adhesions [21]. The inflammatory or reactive involvement of tendons in wrist disease has been described as a cause of spontaneous rupture of damaged extensor tendons [22,23], which is consistent with the author’s experience.

Most of the included studies were not statistically well analyzed, making it difficult to compare results and place them in a common context. Although the scores were not consistently applied across all studies, the studies were conducted over an extended period, during which new individual scores were introduced. These newer scores primarily assess hand functions but also consider the overall situation of the extremity. Quick DASH and ADL/FFbH evaluate the same subjective parameters related to hand usage in daily life. Importantly, limitations in other joints may obscure positive results. Patient-reported outcome measures (PROMs) are useful for scoring, but they do not provide objective measurements for the affected joint or verify the direct impact of limitations on other joints. Owing to the lack of appropriate scores, it is currently difficult to demonstrate a correlation between subjective patient statements and objective assessments. Joint and tendon destruction in rheumatoid arthritis patients does not occur in isolation, even with sufficient therapy according to guidelines. Therefore, it is crucial to consider the patient’s overall condition. The affected extremity joints are usually the first to be impacted, depending on the duration of the disease. Notably, although biologics may often mask activity, this results in low scores both pre- and postoperatively. To improve scores, it is essential to focus on the baseline value and its relation to the results.

On average, the overall situation of the hand is rated as sufficient, with an overall improvement in pain being observed (a reduction in the VAS score of 4.6 points on average). Improvements in wrist load-bearing capacity are accompanied by improvements in test scores in the individual studies. Patient assessment depends on the course of the underlying disease, postoperative care, and patient demands. The individual evaluation of patients by comparing preoperative and postoperative values of the Clayton score demonstrated this. Partial results were observed in the patients. It is important to consider this concerning the progression of the disease. Partial and complete extensor tendon ruptures and slow destruction of the joints are usually functionally tolerated for a long time and seem to compensate one another; therefore, late presentation and operative treatment are often performed. In the cohorts, the wrists had been diseased for an average of 6.4 years before surgical treatment. Other authors report similar results [24]. The average values refer to the totality of the study results, with the arithmetic mean having been calculated.

**Figure 3:**
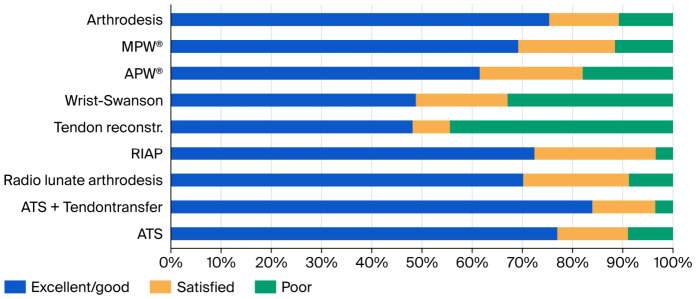
Subjective patient acceptance on a 100% scale. Acceptance is broken down according to the various operations on the hand. ATS = articulo-teno-synovectomy, RIAP = resection-interposition arthroplasty, APW^®^ = anatomical physiological wrist prosthesis; MPW^®^ = modular physiological wrist prosthesis.

To assess the success of the operation for patients, the focus should be on the global function of the wrist (see Figure 3). If intraoperative damage to tendons, especially extensor tendons, is detected along with articular changes, it is crucial to prioritize reconstruction and postoperative therapy. The ability to use the hand effectively in daily life is the main criterion for the subjective evaluation of surgical treatment. Patient satisfaction not only depends on sufficient pain reduction but also appears to be associated with the function of the reconstructed tendons. The tendons are evaluated in conjunction with the everyday usability of the entire extremity. Compared with rheumatic patients, nonrheumatic patients have been found to experience fewer limitations and greater retention of strength. Rydholm et al. reported similar results, highlighting a discrepancy between objectively measured strength and subjective patient assessments of hand function [25].

These observations also support earlier referral for surgical evaluation in patients with persistent synovitis, progressive tendon irritation, ulnar-sided wrist deformity, or loss of active finger extension despite optimized medical treatment. In the biologic era, systemic inflammatory activity may be reduced while local structural damage continues to progress. Consequently, low systemic disease activity scores do not exclude clinically important wrist destruction or tendon compromise. Patient selection is therefore essential. The criteria for indicating surgery for rheumatic wrist conditions remain the same [14,15].

One thing that has changed in the era of biologics is the timing of diagnosis and the start of treatment, and consequently, in many cases, the improved prognosis for the individual. Furthermore, there has been a reassessment of the treatment options for rheumatic joints. Improved implants, early diagnosis of inflammatory conditions, and the initiation of guideline-based basic therapy have transformed the treatment options compared with the prebiological era, which we analyzed and discussed in this manuscript. Outcomes for these patients appear to have improved due to shorter periods of immobilization and early functional exercise. Studies with long-term follow-up of 10 years or more are likely to provide reliable findings in the near future.

With or without insufficient medical treatment, RA continues to follow the same course as it did decades ago. Delayed surgery may result in poorer reconstructive options because tendon quality, carpal height, and bone stock deteriorate over time. Some studies also distinguished postoperative outcomes by the duration and stage of joint damage (see Section 3.4; Section 3.5 MPW^®^). This, together with the clinical implications, forms the basis of the guideline’s recommendation for early surgical intervention in cases of synovitis that persists for more than six weeks despite adequate basic treatment [26].

Revision surgery and patient satisfaction: All the treatments required revision surgery over time. There was a large variation in the time between initial treatment and revision. The longer it had been since the initial operation, the more willing patients were to undergo further treatment. Satisfaction may be the highest in patients with improved grip strength and tendon function.

### Limitations

This study provides valuable information about the results of different treatments for rheumatoid wrist disease. Most of the patients included in the studies had been treated before the introduction of biologics; the results therefore primarily reflect historical treatment strategies. However, some limitations need to be considered. Firstly, the studies summarized here are retrospective, which introduces potential biases such as selection bias. They differ considerably in heterogeneous cohorts and patient characteristics, surgical procedures, follow-up duration, outcome measures, and treatment era. These differences limit direct comparisons and should be discussed more explicitly. It also limits the ability to control for confounding variables, such as differences in postoperative therapies. The main weakness is undoubtedly the lack of statistical comparative analyses and standardized outcome measures used in the different studies. Some of these scores were published after the first trials were conducted and published. A re-examination as part of a new and comprehensive follow-up study is likely to fail because of the limited number of patients, as most of the patients are likely to have died in the meantime.

## 5. Conclusions

Overall, the available evidence indicates that successful treatment of the rheumatoid wrist requires more than correction of joint destruction. The best clinical results are achieved when surgery addresses pain, wrist stability, carpal alignment, and tendon function simultaneously. Future studies should therefore stratify patients according to disease stage, tendon status, bone quality, age, and functional demand, and should combine PROMs with objective measurements of the overall hand function. This would allow more reliable identification of prognostic factors and more precise recommendations for patient selection. Surgeons should be proficient in stage-specific techniques for the surgical treatment of the wrist and the extensor tendons, as summarized in Figure 1. These recommendations are based on experience and consider the professional exchange of views with other clinics treating similar patient groups. As the recommendations are based on studies conducted prior to the use of biological preparations, they still need to be confirmed in current patient groups.

## Figures and Tables

**Table 1:** Results of rheumatoid wrist operations across different studies in points. ATS = articulo-teno-synovectomy, RIAP = resection-interposition arthroplasty, APW^®^ = anatomical physiological wrist prosthesis; MPW^®^ = modular physiological wrist prosthesis, CHR = carpal high ratio acc. to Youme (normal: 0.51 bis 0.57); N = number of patients at time of follow-up.

	N	Clayton Pre	Clayton Post	VAS Pre	VAS Post	LDE Pre	LDE Post	Revision Rate (%)	FFbH Pre	FFbH Post	CHR Pre	CHR Post
ATS	78	-	-	7.6	3	2.1	3.2	13	18.8	22.8		
ATS + tendon-transfer	56	50.3	69.4	7.4	3	2.3	3.3	6.7	-	-		
Radio-lunate arthrodesis	50	-	74.2	7.6	4.2	2.9	3.6	8	18.2	24.9	0.68	0.71
RIAP	28	45	68	6.3	2.6	4	4.5	5	22	26	-	0.4
Extensor tendon	26	49.6	60.1	7.5	2.9	-	-	0	22	20		
Wrist-Swanson	72	47.2	69.4	7.6	4.3	-	-	14.6	21	29.3	0.4	0.32
APW^®^	37	49.2	77.3	-	-	-	-	17.9	20.8	30.5	0.54	0.4
MPW^®^	32	49.3	86.4	7	1.8	-	-	56	-	-		

## Data Availability

The datasets used and/or analyzed during the current study are available from the corresponding author upon reasonable request.

## Abbreviations

The following abbreviations are used in this manuscript:

ADL	Activity of daily life
FFbH	Funktionsfragebogen Hannover—functional questionnaire Hannover
LDE	Larsen, Dale, Eek
RA	Rheumatoid arthritis
PROMs	Patient-reported outcome measures
VAS	Visual analog scale
